# Editorial: Role of membrane-bound and circulating endoglin in disease

**DOI:** 10.3389/fmed.2023.1271756

**Published:** 2023-09-04

**Authors:** Carmelo Bernabeu, Carla Olivieri, Elisa Rossi

**Affiliations:** ^1^Centro de Investigaciones Biológicas “Margarita Salas”, Consejo Superior de Investigaciones Científicas, Madrid, Spain; ^2^General Biology and Medical Genetics Unit, Department of Molecular Medicine, University of Pavia, Pavia, Italy; ^3^Université de Paris, INSERM, Innovative Therapies in Haemostasis, Paris, France

**Keywords:** endoglin, vascular pathology, hereditary hemorrhagic telangiectasia, cancer, hyperglycemia, hypercholesterolemia, hemostasis

## Introduction

Since its discovery and characterization in the early 1990s, a growing body of evidence supports the involvement of endoglin in a broad range of patho-physiological conditions, including physiological and pathological angiogenesis, vascular pathology, preeclampsia, tumor vascularization, hemostasis or tumor malignancy (1–3). This Research Topic draws together a series of reports focusing on different and novel aspects of endoglin on etiology, diagnostics, prognosis, or predictive purposes in several conditions such as hereditary hemorrhagic telangiectasia, endothelial dysfunction, cancer, hyperglycemia, hypercholesterolemia, septic syndrome, and systemic sclerosis.

## Endoglin protein and function

Endoglin is a type 1 transmembrane glycoprotein, encompassing an extracellular region of 561 amino acids, a hydrophobic transmembrane domain, and a 47-residue cytoplasmic tail (4). Approximately 90% of the protein is located within its extracellular region, which upon being targeted, at least by metalloproteases (MMP) MMP-14 and MMP-12, can be shed as a circulating form of endoglin also named as soluble endoglin (sEng) (5, 6). Thus, it is not surprising that the extracellular region has attracted many structural and functional studies (1–3). The extracellular region of endoglin encompasses two distinct domains: (i) a juxtamembrane Zona Pellucida (ZP) domain expanding ~260 amino acids at the C-terminus, with eight conserved cysteine residues and divided into two well-defined subdomains (ZP-C and ZP-N); and (ii) an orphan domain (OD) at the N-terminus, named so due to its lack of significant homology with other protein families (7, 8). These two domains differ from each other in their functional activities. Thus, the OD is involved in the recognition and signaling of members from the transforming growth factor-β (TGF-β) family, like bone morphogenetic protein (BMP)-9 and BMP-10 (8, 9). On the other hand, the ZP domain is involved in cell adhesion through its interaction with integrins of the arginine-glycine-aspartic acid (RGD) subfamily, like α5β1 and αIIbβ3, which recognize the RGD motif located within the ZP-N subdomain of endoglin (3). In addition, the short (14 residues) cytoplasmic domain of endoglin is constitutively phosphorylated in serine and threonine residues and is involved in the organization of the actin cytoskeleton and TGF-β/BMP signaling (10–12).

## Hereditary hemorrhagic telangiectasia

Hereditary hemorrhagic telangiectasia (HHT) is an autosomal dominant vascular dysplasia with a prevalence of ~1:5,000 inhabitants. HHT is characterized by mucocutaneous telangiectases and arteriovenous malformations (AVMs) in the brain lung or liver (13, 14). Heterozygous mutations in several genes are known to cause different variants of HHT (15). Among these, mutations in the endoglin gene (*ENG*) cause HHT1 (MIM #187300) while mutations in activin receptor-like kinase 1 (*ACVRL1* or *ALK1*) result in HHT2 (MIM #600376). It is of note that over 85% of all patients with HHT present with mutations in either *ENG* or *ACVRL1*. A combined syndrome of juvenile polyposis (JP) and HHT was reported to be caused by mutations in *SMAD4* (JP-HHT; MIM 600993), which accounts for ~2% of HHT patients. In addition, rare mutations in *GDF2*, coding for BMP9, a member of the TGF-β family, may cause an HHT-like phenotype (HHT5; MIM 615506). Overall, all the HHT variants share common symptoms, but they differ from each other in the frequency of the specific vascular lesions. All genes involved in HHT code for components of the TGF-β signaling pathway (16) and their targeting in different animal models have focused most of the research and preclinical studies in HHT, as these proteins represent potential therapeutic targets to find novel treatments for the disease. Arthur and Roman have updated the current knowledge about disease mechanisms and potential therapeutic strategies using preclinical animal models of HHT. Recent work has revealed new insights into the cellular and molecular mechanisms causing this disease. Loss of *ENG, ALK1*, or *SMAD4* genes in endothelial cells (ECs) result in AVMs, which can be modulated by the altered directional migration in response to shear stress (17) and increased proliferation of ECs, as well as the crosstalk between ECs and vascular smooth muscle cells, and different angiogenesis factors like VEGF (18) or Angiopoietin 2 (19). To account for the localized and tissue-specific vascular lesions, emerging evidence supports the existence of a second hit (16), including somatic mutations of HHT genes leading to biallelic loss of function (20). Egido-Turrión et al. has analyzed the mechanisms underlying the bleeding in HHT using haplodeficient Eng and Alk1 mouse models. This is a relevant point as HHT is characterized by fragile mucocutaneous telangiectases prone to breaks in the mucosa, causing recurrent and spontaneous epistaxis and gastrointestinal bleeding. However, in addition to the fragility of telangiectases, abnormal hemostasis was reported in HHT1 cellular and animal models owing to an impaired interaction between endothelial endoglin and integrin aIIbβ3 from platelets (21). Given the severity of hemorrhages in some HHT patients, the study of hemostasis appears to be key to finding therapies for this disease. In their exploration of this, Egido-Turrión et al. found that both *Eng*^+/−^ (HHT1) and *Alk1*^+/−^ (HHT2) mouse models present abnormal hemostasis, but that the hemostasis mechanism involved is different between HHT1 and HHT2. While in *Eng*^+/−^ mice an impaired platelet adhesion to endoglin haplodeficient ECs occurs, in *Alk1*^+/−^ mice an overactivation of the fibrinolysis system was observed, as evidenced by elevated levels of D-dimers. These results open new therapeutic avenues to treat bleeding in HHT patients.

## Cancer: squamous cell carcinomas and head and neck neoplasms

Endoglin is highly expressed by tumor-associated vascular endothelium and the expression levels of sEng correlate with poor survival in certain cancer patients (22–24). In addition, endoglin can be expressed by some tumor cells and diverse cell types from the tumor microenvironment (TME) such as cancer-associated fibroblasts (CAFs) or tumor-associated macrophages (TAMs) and lymphocytes (TALs) (25). These characteristics have prompted several lines of investigation to explore endoglin as a potential therapeutic target in cancer. Some of these studies have tested the endoglin neutralizing antibody TRC105 (Carotuximab^®^, Tracon Pharmaceuticals, San Diego, CA, USA), mostly in the context of anti-angiogenic therapies, using a wide variety of preclinical cancer models as well as phase I-III clinical studies of cancer patients (23, 24). Additional studies of cancer development have analyzed the role of endoglin in tumor cells themselves (26). Hakuno et al. analyzed the expression of endoglin in three types of human squamous cell carcinoma (SCC): head and neck (HNSCC), esophageal (ESCC), and vulvar (VSCC) cancers. Analysis of tumor specimens showed that endoglin is selectively expressed by individual SCC cells in tumor nests, while patient-derived HNSCC, ESCC, and VSCC cell lines displayed varying levels of endoglin with high interpatient variation. Endoglin overexpression in SCC cell lines was associated with increased sEng levels, which in turn decreased BMP9 signaling. However, in a ligand-dependent or independent manner, endoglin did not affect the proliferation or migration of the SCC cells. Litwiniuk-Kosmala et al. summarize current data on endoglin expression in head and neck (HN) tumors, which comprise a heterogeneous group of pathologies, including various benign lesions and malignant neoplasms. The reported role of endoglin as a marker in various malignant and non-malignant HN tumors, including HNSCC, salivary gland tumors, paragangliomas, rhabdomyosarcoma, and vestibular schwannomas was briefly reviewed. It is noteworthy that several studies have demonstrated that a high expression of endoglin in these tumor tissues was an independent risk factor that correlates with a lower 5-year overall survival rate.

## Hyperglycemia and hypercholesterolemia

Hyperglycemia and hypercholesterolemia are hallmarks of the so-called metabolic syndrome and risk factors for the development of endothelial dysfunction. Metabolic syndrome is often associated with cardiometabolic disorders such as atherosclerosis, ischemic heart disease, or type II diabetes mellitus (27). Previous studies have shown that sEng combined with hypercholesterolemia aggravates endothelial and vessel wall dysfunction in mouse aorta (2), and sEng combined with a high-fat diet alters NO production. Also, increased levels of sEng have been found in patients with different hyperglycemic conditions, including diabetes mellitus (28) and other hyperglycemic conditions (2). Interestingly, *in vivo* studies have shown that endoglin is critically involved in vascular endothelial pathophysiology, including endothelial dysfunction development (2). Tripska et al. have investigated the effects of the anti-endoglin antibody TRC105 on the development of endothelial dysfunction induced by 7-ketocholesterol or high glucose using human aortic endothelial cells. Their results demonstrate that TRC105-mediated neutralization of endoglin counteracts the hypercholesterolemia- and hyperglycemia-induced endothelial dysfunction, suggesting that endoglin might be a therapeutic target in disorders associated with elevated cholesterol and glucose levels.

## Septic shock and severe COVID-19

Sepsis is a life-threatening condition that arises from a dysregulated host response to infection. It can progress, first to severe sepsis, and then to septic shock, leading to a blood pressure drop and multiple organ damage and failure (29, 30). The World Health Organization (WHO) has recognized sepsis as a major cause of preventable morbidity and mortality worldwide, highlighting the common and nosocomial pathogens involved in this condition, and alarming pathogen resistance to antibiotics (31). Sepsis is associated with dysregulation of hemostasis and vascular reactivity, likely due to EC dysfunction and inflammation, processes in which endoglin and sEng are involved (2, 32). Interestingly, sEng is a biomarker of several pathologic conditions, including sepsis, where its increased levels are associated with endothelial dysfunction. Indeed, patients with septic shock present 1.5-fold higher levels of sEng compared to healthy individuals (33). More recently, sEng was found to be independently associated with complicated course and acute renal dysfunction in pediatric septic shock (34). Tomášková et al. analyzed the prognostic value of sEng in patients with septic shock and severe COVID-19. In patients with COVID-19, the main clinical manifestation observed was acute respiratory distress syndrome (ARDS), and sEng did not predict mortality or correlate with markers of organ dysfunction. By contrast, in septic shock, sEng levels were significantly higher in patients with early mortality and correlated with signs of circulatory failure. These results suggest that sEng could be used for the early identification of patients with severe endothelial dysfunction who would benefit from an individualized endothelium-targeted therapy.

## Systemic sclerosis

Systemic sclerosis (SSc), also known as scleroderma, is a group of rare autoimmune diseases that affects the connective tissue with widespread vasculopathy and inflammation. SSc is associated with an excess of collagen fiber deposition, leading to hardening and tightening of the skin. Other common symptoms include calcinosis, skin telangiectases, pulmonary arterial hypertension (PAH), fatigue, weight loss, musculoskeletal inflammation, and gastrointestinal involvement (35). Unfortunately, the etiology of SSc is still poorly understood. A pathogenic role TGF-β signaling pathway has been postulated in this disease due to its stimulatory properties on fibroblasts and extracellular matrix production. In this context, several lines of evidence suggest the potential involvement of endoglin in SSc: (i) endoglin is involved in TGF-β signaling modulating ECM production, which is critically involved in SSc; (ii) the existence of common symptoms (ie, telangiectases) between SSc and HHT1 (caused by mutations in *ENG*); and (iii) the reported role of sEng as a biomarker of several inflammatory conditions similar to SSc. Accordingly, the possible link between endoglin and SSc has been analyzed in over thirty different publications, which have now been reviewed by Grignaschi et al. following the Preferred Reporting Items for Systematic Reviews and Meta-Analyses (PRISMA) guidelines. This systematic review revealed a dysregulated expression of endoglin in SSc-affected cells and tissues, while clinical studies support the notion that levels of circulating sEng correlate with different disease phenotypes, suggesting that endoglin plays a role in disease-related mechanisms, including the involvement in different TGF-β-stimulated pathways that can be crucial in SSc pathogenesis and progression.

## Conclusions

In recent years emerging aspects of membrane-bound endoglin and circulating endoglin on etiology, diagnostics, or prognosis in several conditions have been reported. The collection of articles on this Research Topic encompasses new findings and major advances in disease mechanisms as well as scientific and clinical research on hereditary hemorrhagic telangiectasia, cancer, hyperglycemia, hypercholesterolemia, septic shock, severe COVID-19, and systemic sclerosis. Further translational studies on endoglin are needed in order to integrate the results of these investigations into clinical practice.

## Author contributions

CB: Conceptualization, Formal analysis, Investigation, Supervision, Validation, Visualization, Writing—original draft, Writing—review and editing. CO: Formal analysis, Investigation, Supervision, Validation, Visualization, Writing—review and editing. ER: Formal analysis, Investigation, Supervision, Validation, Visualization, Writing—review and editing.
